# Interleukin-17 and Its Implication in the Regulation of Differentiation and Function of Hematopoietic and Mesenchymal Stem Cells

**DOI:** 10.1155/2015/470458

**Published:** 2015-04-27

**Authors:** Slavko Mojsilović, Aleksandra Jauković, Juan F. Santibañez, Diana Bugarski

**Affiliations:** Laboratory for Experimental Hematology and Stem Cells, Institute for Medical Research, University of Belgrade, Dr. Subotića 4, P.O. Box 102, 11129 Belgrade, Serbia

## Abstract

Adult stem cells have a great potential applicability in regenerative medicine and cell-based therapies. However, there are still many unresolved issues concerning their biology, and the influence of the local microenvironment on properties of stem cells has been increasingly recognized. Interleukin (IL-) 17, as a cytokine implicated in many physiological and pathological processes, should be taken into consideration as a part of a regulatory network governing tissue-associated stem cells' fate. This review is focusing on the published data on the effects of IL-17 on the properties and function of hematopoietic and mesenchymal stem cells and trying to discuss that IL-17 achieves many of its roles by acting on adult stem cells.

## 1. Introduction

Adult stem cells are present in virtually all tissues of a developed organism and are involved in tissue homeostasis and regeneration. Due to their remarkable properties, adult stem cells have a great potential applicability in regenerative medicine, as a support of hematopoiesis, and in immunomodulation [1–3]. Some of the adult stem cells, as hematopoietic stem cells (HSCs), are already in the clinical use for decades [4], while others are still in preclinical and clinical trials (https://www.clinicaltrials.gov/). Despite the significant progress in understanding the nature, functions, and mechanisms of action of adult stem cells, there are still many ambiguities and unresolved issues necessary for their effective and safe application [5, 6]. On the other hand, there is increasing number of reports showing the role of local stem cells in numerous diseases, such as inflammatory diseases and cancer [7, 8]. It is now well established that inflammatory milieu has major influence on stem cells [9]. Interleukin (IL-) 17 with its roles in many physiological and pathological processes, such as inflammation, immune response, and regulation of hematopoiesis [10], is an appreciable candidate for a major factor in guiding stem cells' fate. There is now considerable amount of data showing the effects of IL-17 on proliferation and function of adult stem cells. The purpose of this review is to analyze the published data, focusing on the impacts of IL-17 on the properties and fate of hematopoietic and mesenchymal stem cells, and to discuss that IL-17 accomplishes many of its roles in homeostasis and diseases by acting on stem cells.

## 2. Interleukin-17

IL-17 is a prototypic and the most extensively studied member of the newest cytokine family comprising six members (IL-17A-F). IL-17 is mainly produced not only by the new subclass of helper T cells, Th17, but also by other cells, such as innate immune cells, CD8^+^ T cells, B cells, and MSCs [11–14]. A number of cytokines, including TGF-*β*, IL-1*β*, IL-6, IL-21, and IL-23, enable and control Th17 programming by activation of ROR*γ*t and STAT3 transcription factors [15, 16].

IL-17 receptor family consists of five homologous type I transmembrane protein receptors: IL-17RA to IL-17RE. IL-17Rs, and IL-17RA in particular, as being considered a common signaling subunit of IL-17R family, are ubiquitously present and their expression is demonstrated in virtually all tested cells [17–19]. Upon IL-17A binding, heteromeric complex of IL-17RA and IL-17RC is formed, triggering the initiation of downstream signaling events. Activated IL-17RA through a conserved SEF/IL-17R (SEFIR) domain interacts with the adaptor molecule Act1 to start several downstream signaling processes. One of them engages TRAF6 and involves three major downstream pathways: nuclear factor-*κ*B (NF-*κ*B), mitogen-activated protein kinase (MAPK), and CCAAT/enhancer-binding protein (C/EBP) pathways. Others include IKKi-TRAF2-TRAF5-dependent cascade, PI3K, and JAK-STAT pathways. Besides SEFIR, a second functional domain, C/EBP*β*-activation domain (CBAD), exists on IL-17RA C-terminal region [19–21].

IL-17 signaling induces the expression of proinflammatory cytokines, chemokines, antimicrobial peptides, growth factors, tissue remodeling enzymes, and other secondary mediators in target cells [10]. IL-17 alone often induces weak response, but it may synergize with other cytokines, like TNF-*α*, to enhance and prolong proinflammatory responses [17, 20–23].

The role of IL-17 and Th17 pathway in numerous physiological and pathological processes is being increasingly recognized. IL-17 has its role in the homeostasis, particularly in the regulation of bone metabolism and hematopoiesis, as well as in the pathogenesis of numerous autoimmune and inflammatory diseases [24–29].

## 3. Stem Cells

Stem cells are primitive, nonspecialized cells with the ability to self-renew and to differentiate into one or more specialized cell types [1, 2]. Owing to these key features, stem cells have been envisaged as a promising tool for regenerative and cell-based therapies. Stem cells are present during the entire ontogenesis of an individual, starting from a totipotent zygote, through pluripotent embryonic stem cells, and continuing all the way to the adulthood, progressively decreasing their multilineage differentiation potential to multipotent, oligopotent, and unipotent adult or somatic stem cells [3].

Although the concept of stem cells was introduced even in the late 19th century [30], it was not until 1960s that James Till and Ernest McCulloch described hematopoietic stem cells in bone marrow and demonstrated the key characteristics of stem cells, self-renewal and multilineage differentiation potential [31]. The existence of nonhematopoietic, bone-forming cells inside bone marrow was first reported in late 1960s and early 1970s by Friedenstein et al. [32, 33]. Later, other researchers demonstrated the* in vitro* and* in vivo* multilineage differentiation capacities of these cells which they varyingly dubbed stromal stem cells or mesenchymal stem cells (MSCs) [34–36].

A great burst of interest in stem cell research was made after the isolation of stem cells from mouse embryo in 1981 [37] and much later from human blastocyst in 1998 [38]. However, due to ethical issues, along with numerous technical difficulties in isolating, cultivating, and controlling* in vivo* differentiation path of these embryonic stem cells, adult stem cells came back into the focus of interest. The real breakthrough in stem cell research was made in 2006, when Takahashi and Yamanaka induced pluripotency in somatic cells by the transduction of four key genes [39]. This not only brought about the new potential modalities of stem cell exploitation, but also set a new groundwork for research in embryology and cell biology.

### 3.1. Hematopoietic Stem Cells

Hematopoietic stem cell (HSC) is the first and the most comprehensively studied model of an adult stem cell [1, 40]. HSCs are multipotent adult stem cells that give rise to all the hematopoietic lineages. They are able to undergo self-renewing divisions for a lifetime of an individual. A single HSC can reconstitute the entire hematopoietic system of a lethally irradiated mouse [41]. By further differentiation, HSCs give rise to more and more specialized progenitors. At the same time, HSCs keep the constant stem cell pool by asymmetric division, whereby HSC produces one identical daughter stem cell and one that follows differentiation path. In case of need (e.g., during development or injury), HSCs can divide by symmetrical division, to produce only identical HSCs, and thus replenish stem cell pool [42]. HSCs reside in endosteal niche in bone marrow where they receive informative cues by cell-to-cell contacts, cell-matrix interactions, and soluble factors that are necessary for their survival, self-renewal, differentiation, and migration, such as SCF, Flt-3 ligand, M-CSF, G-CSF, GM-CSF, IL-3, IL-6, IL-7, IL-8, IL-11, EPO, TPO, LIF, RANKL, SDF-1, N-cadherin, and many others. They are under concerted influence of environmental factors, like neighboring cells, extracellular matrix, endocrine, paracrine, and neural signals, as well as physical and metabolic stimuli [43, 44].

### 3.2. Mesenchymal Stem Cells

Mesenchymal stem/stromal cells (MSCs) are multipotent stromal cells first discovered in bone marrow as cells capable of forming hematopoietic microenvironment after heterotopic transplantation into nude mice [33]. Now, it is recognized that MSCs are present in almost all tissues [45] and are responsible for the maintenance of tissue homeostasis, regeneration, and repair. Beyond their role in regular cell turnover in connective tissues and tissue repair after injury, MSCs have a major role in the control of tissue inflammation, as well as in the formation of HSC niches and regulation of hematopoiesis [46–55]. In response to certain factors, MSCs can be mobilized and recruited to the site of injury, chronic inflammation, or tumor [56, 57]. For all these remarkable properties and since they are easily accessible from diverse adult tissues, MSCs are envisaged as a great tool for cell and gene therapy [50, 58]. However, notwithstanding the plethora of* in vitro* research and data showing characteristics and function of MSCs, the true nature and origin of MSCs* in vivo* is still a matter of debate [5, 6, 59]. Numerous problems hinder MSC research, such as their considerable heterogeneity and lack of specific markers for prospective isolation, as well as changes in their properties during* in vitro* cultivation [3, 59–62]. In order to standardize criteria for the identification of MSCs, the International Society for Cellular Therapy provided a set of minimum criteria for defining human MSCs, which include plastic adherence, a set of positive and negative markers, and three-lineage differentiation capacity [63].

## 4. Role of IL-17 in Hematopoiesis and Hematopoietic Stem/Progenitor Cells Regulation

IL-17 has significant role in hematopoiesis [27]. Its role in granulopoiesis has been shown in some of the first reports on this cytokine [64, 65]. IL-17 induced proliferation of human bone marrow CD34^+^ cells* in vitro* and their differentiation into granulocytes, albeit only in the presence of fibroblasts [64]. Also, IL-17 induced increase in the number of committed hematopoietic precursors in the coculture of CD34^+^ cells and MSCs from human bone marrow [66]. Our group showed increase in hematopoietic progenitors in total mouse bone marrow cell cultures after treatment with IL-17 [67]. In each case, the effects of IL-17 on hematopoietic stem/progenitor cells seemed to be indirect, via secondarily induced mediators, such as G-CSF, IL-6, and erythropoietin. This stimulatory effect of IL-17 on hematopoiesis, especially on myelopoiesis, has been supported by* in vivo* studies, and, likewise, it has been dependent on hematopoietic cytokines, G-CSF and IL-6, as well as on transmembrane form of stem cell factor [65, 67–71]. The* in vivo* expression of IL-17 in an experimental model of adenovirus-mediated gene transfer of the murine IL-17 cDNA induced a profound stimulation of both bone marrow and splenic granulopoiesis and led to expansion of myeloid hematopoietic stem and progenitor cells and neutrophilia [65, 69]. Moreover, a routine complete blood count analysis of transgenic mice overexpressing IL-17 revealed an anemia-like phenotype, along with increase in granulocyte number in peripheral blood, spleen, and bone marrow [72]. In a different experimental approach, IL-17 recombinant protein injected in a normal mouse elicited a cascade of biological changes, affecting primarily the cells of granulocytic lineage, as well as the levels of secondary mediators released, in both murine bone marrow and spleen [67, 70, 71, 73]. On the other hand, IL-17RA knock-out mice show normal baseline hematopoiesis, with peripheral hematological parameters and clonogenic progenitor assay scoring comparable to their normal littermate controls [74]. The role of IL-17 in hematopoiesis is largely dependent on specific tissue microenvironment, since there is more profound stimulation of both myeloid and erythroid progenitors in spleen than in bone marrow [65, 71, 73]. The effect of IL-17 is also dependent on the lineage and differentiation status of the progenitors, as well as on the physiological status of the organism. Namely, in healthy mouse bone marrow, IL-17 stimulates myeloid progenitors (colony-forming unit-granulocytic/monocytic, CFU-GM) and early stage erythroid progenitors (burst-forming unit-erythroid, BFU-E) but inhibits late stage erythroid progenitors (colony-forming unit-erythroid, CFU-E) [67, 75, 76]. However, in case of disturbed hematopoiesis, such as upon radiation or infection, the response to this cytokine is significantly altered [67, 74, 76, 77], indicating that the action of IL-17 on hematopoiesis is deeply reliant on the microenvironment and the induction of other regulators, but also that it primarily acts in response to a distress, rather than as a baseline homeostatic factor.

**Figure 1:**
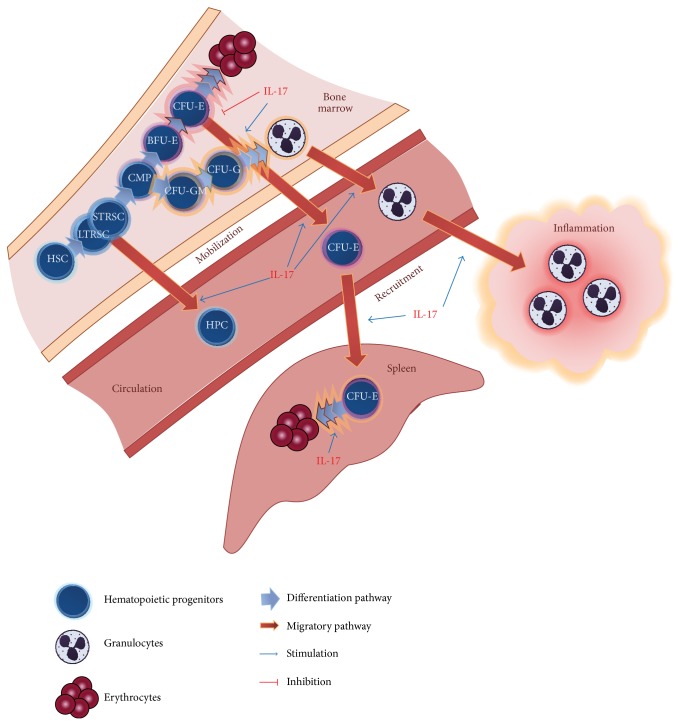
Effects of IL-17 on hematopoietic cells. In bone marrow, IL-17 stimulates granulopoiesis and downregulates erythropoiesis through inhibition of late stage erythroid progenitors, CFU-E. At the same time, IL-17 stimulates mobilization of hematopoietic progenitors and mature granulocytes into circulation. Through secondarily induced chemotactic factors, IL-17 stimulates recruitment of granulocytes into a site of inflammation. It also stimulates erythropoiesis in spleen, by mobilizing erythroid progenitors from bone marrow to spleen and stimulating splenic CFU-E differentiation. HSC: hematopoietic stem cell; HPC: hematopoietic progenitor cell; LTRSC: long-term repopulating stem cell; STRSC: short-term repopulating stem cell; CMP: common myeloid progenitor; CFU-GM: colony-forming unit-granulocytic-monocytic; CFU-G: colony-forming unit-granulocytic; BFU-E: burst-forming unit-erythroid; CFU-E: colony-forming unit-erythroid.

Another putative role of IL-17 in preserving the required level of hematopoietic and immune system response to stress signals during injury and inflammation is its potential to affect mobilization of hematopoietic stem cells (Figure 1). In the mouse model of IL-17-overexpression using adenovirus-mediated gene transfer, IL-17 stimulated the recruitment of both stem cells with short- and long-term reconstituting capacity, and these cells successfully rescued lethally irradiated mice [78]. Results from our experimental model demonstrated that multiple application of recombinant mouse IL-17 mobilized erythroid progenitors to peripheral blood and suggested the possibility that it relocated core erythropoiesis from bone marrow to spleen [73]. However, the exact role and mechanisms of action of IL-17 in mobilization of hematopoietic stem cells warrant further studies.

## 5. Role of IL-17 in Mesenchymal Stromal/Stem Cells Regulation

It was suggested, even in the beginning of the study of IL-17, that its mode of action is primarily by inducing diverse soluble or membrane-bound factors, such as IL-6, G-SCF, GM-CSF, SCF, NO, prostaglandins, chemokines, and other inflammatory and growth factors in stromal and other accessory cells, like macrophages and endothelial and epithelial cells [10, 17, 18, 64, 79–82]. IL-17RA is expressed at particularly high levels on stromal cells, including MSCs, both in human and in mice [17, 83–85]. It is thus reasonable to consider MSCs as a target of IL-17 action. Because of their important role in tissue regeneration and homeostasis, as well as their availability from numerous tissues, MSCs are envisaged as a promising tool for regenerative and cell therapy. Still, there are numerous obscurities concerning their biology, especially the influence of local microenvironment on their differentiation and function. The roles of inflammatory cells and cytokines in MSC-governed tissue regeneration, regulation of hematopoiesis, and immunomodulation have already been demonstrated [86–89]. However, the data regarding the influence of IL-17 on MSCs proliferation, differentiation, and function are relatively scarce and are only recently being reported. IL-17 increased the frequency and the average size of CFU-F (colony-forming units-fibroblast) derived from murine and human bone marrow, as well as the proliferation of murine and human bone marrow-derived MSCs, in a dose-dependent manner [84, 85, 90]. In human MSCs, this effect was dependent on the generation of reactive oxygen species (ROS) by TRAF-6 and Act1-mediated activation of NADPH oxidase 1 (Nox1) and subsequent activation of MEK-ERK MAPK pathways [84]. In the mouse model, both p38 and ERK MAPKs were involved in IL-17-induced proliferation of bone marrow-derived MSCs [85].

**Figure 2:**
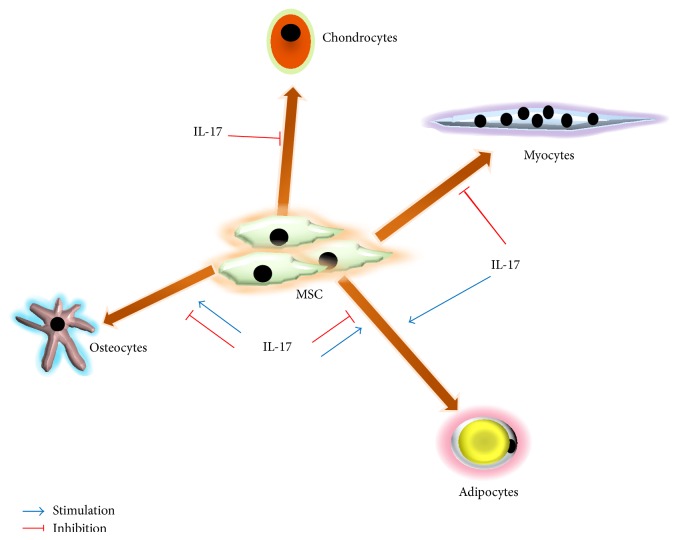
Effects of IL-17 on the differentiation of MSCs. IL-17 stimulates or inhibits MSCs differentiation into osteocytes, adipocytes, chondrocytes, and myocytes, depending on the host, origin of MSCs, and microenvironmental factors.

The data concerning the influence of IL-17 on differentiation of MSCs are much more ambiguous and depend on species and tissue of origin (Figure 2). Namely, in human bone marrow-derived MSCs, IL-17 was shown to enhance osteogenic differentiation [84, 91] and to inhibit adipocyte differentiation [92]. The latter was, at least partially, mediated through induction of cyclooxygenase-2 expression and a consequential increase of antiadipogenic prostaglandin E_2_ [92]. IL-17 also inhibited chondrogenesis of human MSCs through the suppression of protein kinase A activity and a consequent decrease in SOX9 phosphorylation, a major chondrogenesis transcription factor [93]. In our experiments, using mouse bone marrow-derived MSCs, IL-17 did not affect their differentiation potential to osteoblasts or adipocytes [85]. However, IL-17 suppressed osteogenic differentiation and bone formation of mouse bone marrow-derived MSCs, via I*κ*B kinase and NF*κ*B [94], and of rat osteoblast precursor cells [95]. On the other hand, in mouse multipotent cell line, C2C12, IL-17 led to their transdifferentiation into adipocytes through C/EBP-*β*-mediated PPAR*γ* activation, a transcription factor crucially involved in adipogenesis [96]. On the same cell line, our group showed that IL-17, through ERK1,2 MAPK activation, switches the balance of differentiation of these multipotent myoblast progenitors from myogenic to osteogenic lineage [97]. Whether these differences are a consequence of species- or tissue-specific properties of different MSCs or a result of interplay of different microenvironmental factors is not elucidated yet. Kim et al. showed different IL-17 receptor subtype expression profile of rat calvarial osteoblast progenitors (primarily IL-17RB, IL-17RD, and IL-17RE) [95], compared to human bone marrow-derived MSCs (primarily IL-17RA and IL-17RC) [84], which may imply different affinity for IL-17A family member, as well as different downstream signaling events. Furthermore, Huang et al. [90] suggested that IL-17 for its* in vivo* effect on CFU-F expansion requires additional cofactors, since it was only observed after application of myeloablative dose of radiation. IL-17 is well known for its cooperative action in combination with other growth and inflammatory factors, which gave it the epithet of “fine-tuning cytokine” [23]. Osta et al. demonstrated synergistic interaction between IL-17 and TNF-*α* on bone matrix formation by human MSCs [91]. The specific tissue and organism requirements, like injury, inflammation, or aging, drive different MSCs responses [9, 98, 99], and IL-17, as part of the complex cytokine network, is proving to be one of the key players governing these responses.

In addition to the role in differentiation of MSCs, IL-17 was also shown to be involved in migration and mobilization of MSCs. Huang et al. reported that IL-17 stimulates migration and motility of human bone marrow-derived MSCs [84]. Moreover, our group demonstrated that IL-17 induces urokinase type-plasminogen activator (uPA) in peripheral blood MSCs, increasing their* in vitro* motility, endothelial adhesion, and transendothelial migration, indicating a possible role of IL-17 in MSCs mobilization and recruitment to damaged tissues [100]. As a proteolytic enzyme and activator of plasmin, and by activating intracellular signaling events, uPA is implicated in migration, adhesion, proliferation, and differentiation of various cell types [101, 102]. Conversely, besides inhibiting myogenic and promoting osteogenic differentiation of C2C12 cell line, IL-17 also inhibited their migration by inhibiting uPA expression through p38 MAPK activation [103].

The immunomodulatory function of MSCs is very prospective for therapeutic exploitation. However, there are still many ambiguities, and various factors influence their immunoregulatory potential. Numerous studies, both* in vitro* and* in vivo*, imply notable immunosuppressive properties of MSCs and effects on almost all immune cells, including T cells, B cells, NK cells, NKT cells, regulatory T cells, dendritic cells, and macrophages [54, 104, 105]. On the other hand, there are reports of antigen-presenting and immunostimulatory role of MSCs [106–112]. It is now widely accepted that MSCs require a “licensing” step, attained in an inflammatory setting, in order to gain their immunocompetence and exert their immunosuppressive effect [54, 88]. Even though there are numerous factors involved in directing immunomodulatory function of MSCs, there are not many reports of the role of IL-17 in this context. Our experiments on mouse bone marrow MSCs did not show any influence of IL-17 on immunomodulatory potential of these cells (*unpublished results*). Our preliminary results on human peripheral blood MSCs and periodontal ligament-derived MSCs also did not provide any conclusive data on this stand. We can only speculate that the presence of IL-17 alone is not a sufficient signal to modify MSCs immunomodulatory potential. Indeed, a recent report showed that IL-17 enhances immunosuppressive effects of IFN-*γ* and TNF-*α* on mouse bone marrow-derived MSCs, both* in vitro* and* in vivo*, in an iNOS-dependent manner [113]. Conversely, a study using synovium-derived MSCs from rheumatoid arthritis patients showed that IL-17, as well as TNF-*α*, alone and in combination, stimulated proliferation of synovial T cells in the presence of these mesenchymal cells [114]. Further studies are necessary to determine the exact role of IL-17 in MSC-mediated immunomodulation.

## 6. Concluding Remarks

The role of inflammatory cytokines in regulation of hematopoiesis, both steady state and stress-induced, has been well documented [115, 116], and IL-17 is recognized as a pivotal cytokine linking immune and hematopoietic system [27]. It can enable the switch in hematopoietic cell production from the erythroid to the granulocyte lineage by stimulation of proliferative granulocytes and inhibition of CFU-E in bone marrow when there is a demand for enhanced defenses, during inflammation or infection. At the same time, its stimulatory effect on erythroid progenitors in mouse spleen could be sufficient to maintain efficient erythropoiesis.

Immune cells, through the production of cytokines and growth factors, influence MSCs mobilization, recruitment, and regenerative and immunomodulatory capacity [9, 54, 89]. It is established, for both exogenously applied and endogenous circulating MSCs, that these cells preferentially engraft at the site of inflammation* in vivo *[54, 56]. Our* in vitro* data suggest that IL-17 can be a recruitment signal for peripheral blood MSCs to migrate and engraft into inflamed tissue in an uPA-activity-dependent way [100]. uPA is known for its role in migration and mobilization of human MSCs from bone marrow, contributing to migration into wounded tissue [117], as well as in transendothelial migration of neutrophils [118]. IL-17, in association with TNF-*α*, has also been shown to affect activation of endothelial cells and to increase neutrophil transmigration and the expression of adhesion molecules and chemokines in human vascular endothelial cells (HUVEC), suggesting that IL-17 facilitates transendothelial migration through induction of endothelial inflammation, as well [119, 120].

Abnormal IL-17 expression and presence of Th17 cells in inflamed tissues have been a hallmark of many inflammatory and autoimmune diseases, including rheumatoid arthritis, inflammatory myopathies, inflammatory bowel disease, multiple sclerosis, and psoriasis [26, 28, 29]. Elucidation of its role in modulation of MSCs function could provide a new perspective on pathogenic mechanisms that underlie these diseases and possibly propose some new therapeutic targets. MSCs also have their role in inflammatory diseases. Local inflammatory microenvironment in rheumatoid arthritis, for example, affects MSCs regenerative and immunomodulatory properties, as well as their survival characteristics [121]. Although* in vitro *data have shown that synovium-derived MSCs isolated from rheumatoid arthritis patients display immunosuppressive properties comparable to the same MSCs isolated from healthy donors, upon treatment with IL-17 and/or TNF-*α* these cells increased proliferation of PHA-stimulated T cells in coculture [114]. This report could explain some of discrepancies observed between* in vitro* and* in vivo* effects of murine mesenchymal stem cells on T cell proliferation and collagen-induced arthritis [122]. Effects of inflammatory factors, such as IFN-*γ* and TNF-*α*, in rheumatoid arthritis have also been proven to negatively influence the osteogenic differentiation of MSCs and to induce their apoptosis [123]. The impact of IL-17 on differentiation potential of MSCs in inflammatory diseases is not so clear. Huang et al. demonstrated that IL-17 not only stimulated osteogenic differentiation of human bone marrow-derived MSCs, but also induced expression of M-CSF and RANKL, crucial factors for osteoclast differentiation and survival [84]. However, it is not known how IL-17 would influence MSCs osteogenic differentiation in the setting of rheumatoid inflammatory milieu. Nonetheless, the pathogenic role of IL-17 in rheumatoid arthritis is clearly established, promoting both inflammation and bone destruction [28, 124]. On the other hand, in C2C12 myoblast cell line, IL-17 inhibited their myogenic differentiation, migration, and myotube formation, via inhibition of uPA expression [97, 103], indicating a rationale for the proposed role of this cytokine in pathogenesis of inflammatory myopathies [29, 125].

Conversely, MSCs reduce the capacity of Th1 and Th17 cells to produce IFN-*γ* and IL-17, respectively, and inhibit Th17 cell differentiation and function, inducing regulatory T cell phenotype [126, 127]. We can speculate that increased levels of IL-17 in inflamed tissues may act as a tropic signal for MSCs, as shown for peripheral blood MSCs [100]. These cells may in turn regulate IL-17 production in a negative feedback to control excessive inflammation. Liu et al. hypothesized that MSC-based tissue regeneration could be improved by modulating recipient T cell response [123]. They systemically infused regulatory T cells and markedly improved MSC-based bone regeneration and repair of calvarial defect in mice. Alternatively, pharmacological inhibition of IFN-*γ* and TNF-*α* by local administration of aspirin produced similar results. This kind of intervention with small molecule inhibitors provides a great therapeutic possibility for treating inflammation-induced tissue injuries by reducing inflammation on one side and enhancing reparative potential of MSCs on the other. Similarly, Chang et al. presented potential for IKKVI, a small molecule inhibitor of I*κ*B kinase, in regeneration of craniofacial bone defect in mice [94].

However, prospective therapeutic approach necessitates additional studies to further elucidate the exact role of IL-17 as a part of an intricate network of regulators governing stem cells fate.
